# Force-regulated robotic diffuse reflectance spectroscopy scanning

**DOI:** 10.1117/1.JBO.31.12.123306

**Published:** 2026-10-08

**Authors:** Kaizhong Deng, Zhangxi Zhou, Maxime Giot, Ioannis Gkouzionis, Christopher Peters, George Mylonas, Daniel Elson

**Affiliations:** a Imperial College London, Institute of Global Health Innovation, Hamlyn Centre for Robotic Surgery, London, United Kingdom; b Imperial College London, Department of Surgery and Cancer, London, United Kingdom

**Keywords:** robot-assisted imaging, medical imaging, robotics system, diffuse reflectance spectroscopy, clinical photonics

## Abstract

**Significance:**

Intraoperative discrimination between malignant and benign tissue is essential for achieving complete tumor resection while preserving healthy structures. Robotic diffuse reflectance spectroscopy (DRS) enables automated intraoperative tissue assessment, but unregulated probe–tissue contact forces introduce spectral artifacts and risk tissue damage, limiting clinical translation.

**Aim:**

We present and evaluate an autonomous robotic DRS scanning system incorporating real-time contact force regulation to enable safe, repeatable, and large-area *ex vivo* tissue assessment.

**Approach:**

A serial robot manipulator equipped with a fiber-optic DRS probe, load cell, and camera employed hybrid visual servoing and force feedback control, maintaining 0.08 N target force with <0.30  N peak limits. Performance was evaluated on *ex vivo* bovine and ovine tissues including scanning safety, force regulation versus expert operators, spectral consistency across six novice operators, and tissue classification using support vector machines.

**Results:**

The system achieved 100% and 93% scan completion rates on bovine and ovine tissues, respectively, without visible tissue damage. Autonomous operation enabled 46 continuous trials over ∼30  min without human intervention. Mean applied force (0.079 N) matched manual sampling (0.080 N) with reduced variance and lower peak forces. Robotic spectral acquisitions aligned with manual measurements, yielding mean inter-observer spectral angles of 1.43 deg, comparable to inter-human operator variability. Tissue classification demonstrated high discriminatory accuracy in bench-top validation.

**Conclusions:**

Force-regulated robotic DRS enables standardized, autonomous tissue scanning with expert-level spectral consistency, advancing spectroscopic sensing toward reliable automated surgical margin assessment.

## 1 Introduction

Achieving complete tumor resection while preserving healthy tissue remains a fundamental objective in oncological surgery, directly correlating with enhanced disease-free survival outcomes.^1^ For this reason, accurate intraoperative differentiation of tissue is crucial to ensure the resection margins are tumor-free. This requirement has intensified the demand for real-time intraoperative tissue assessment technologies capable of providing immediate tissue characterization without dependence on time-intensive histopathological analysis.

Several optical sensing modalities have demonstrated substantial potential for enabling real-time, noninvasive tissue classification, including diffuse reflectance spectroscopy (DRS),^2^ Raman spectroscopy,^3^ and microonfocal endoscopy.^4^ DRS is a well-established technique^5^ for tissue assessment that has demonstrated high accuracy for differentiating normal and cancerous tissues in *ex vivo* upper^2^ and lower^6^ and *in vivo*^7^ gastrointestinal tissues. The technique offers several compelling advantages, including cost-effectiveness, high diagnostic accuracy, intuitive operation, and real-time spectral analysis capabilities.

The operational principle of DRS involves illuminating tissue with broadband white light and analysing the backscattered photons that have undergone multiple scattering and absorption events within the tissue.^8^ Spectral analysis of the acquired reflectance data enables quantitative extraction of tissue optical properties, thereby facilitating compositional assessment and robust differentiation between malignant and healthy tissue types. DRS systems are typically implemented as handheld fiber-optic probes, enabling surgeons to perform point measurements at targeted anatomical locations. However, the technique requires stable probe-tissue contact to ensure optimal signal acquisition and measurement repeatability.

Previous studies on robot-assisted spectroscopy have demonstrated success on *ex vivo* specimens but require meticulous preparation protocols, including microscope slide mounting^9^ or sectioning into discrete fragments.^10^ Several systems have been introduced to facilitate large-scale sample examination, including large tissue samples^11^ and organ phantoms.^12^ However, these approaches did not incorporate systematic probe-tissue contact management strategies.

Contact conditions have been demonstrated to substantially influence DRS spectral characteristics, with applied contact pressure serving as a critical parameter that significantly modulates spectral measurements.^13^^,^^14^ Studies suggest that this effect exhibits non-linear behaviour.^15^ Previously, a self-calibrated pressure-sensing probe was developed by integrating a force sensor into the probe handle, allowing tissue physiology to be assessed using DRS and contact force simultaneously;^16^ however, it lacks a means of controlling the contact force, leaving this responsibility to the operator. A hydraulically powered DRS probe was designed as a modular unit within an MRI-compatible system, measuring the contact force along the coaxial probe axis at the handle and regulating it accordingly;^17^ however, it is fixed to a base and therefore incapable of movement. A wide-field DRS array imaging system enables precise feedback control of contact pressure using a custom-built motorized system for translational movement;^18^ however, it is unsuitable for scanning large areas of tissue due to its limited workspace. A force-adaptive control system was previously introduced for probe-based autofluorescence spectroscopy on an articulated robotic endoscope, enabling the operator to maintain a constant contact force with tissue.^19^ Although force regulation has been achieved in these systems, extending them to large-area tissue scanning remains a challenge.

Recently, a robotic-assisted system for DRS scanning has been developed with contact condition control.^20^ Instead of directly regulating force, this system maintains consistent indentation depth as a proxy for contact condition management. Although this approach has demonstrated success in DRS data acquisition, a critical limitation of indentation-based control is the potential for tissue damage when applied to soft or fragile biological structures, as the predetermined indentation depth may exceed the elastic deformation limits of delicate tissues. Furthermore, force-dependent spectral distortions can introduce significant variance in DRS sampling,^21^ compromising the reproducibility and accuracy of spectral measurements. This limitation means the system cannot ensure safe tissue handling across diverse anatomical structures, potentially reducing its overall reliability.

In this study, we evaluate a framework for a robotic-assisted autonomous DRS scanning of tissue specimens with integrated contact force control. It utilized a hybrid visual servoing approach with force feedback to guarantee the safe manipulation of the soft tissue, as shown in Fig. 1. With the effective contact force control, the system limits sudden force peaks above 0.3 N and maintains stable probe–tissue contact, thereby helping to preserve specimen integrity during prolonged use with repeated scanning tests in our experiments. Furthermore, in an exemplar biological tissue classification task, the acquired spectra yield a classification accuracy of 93.8%, highlighting the potential of the proposed system as a reliable tool for intraoperative tissue assessment without requiring additional tissue preparation steps.

**Fig. 1:**
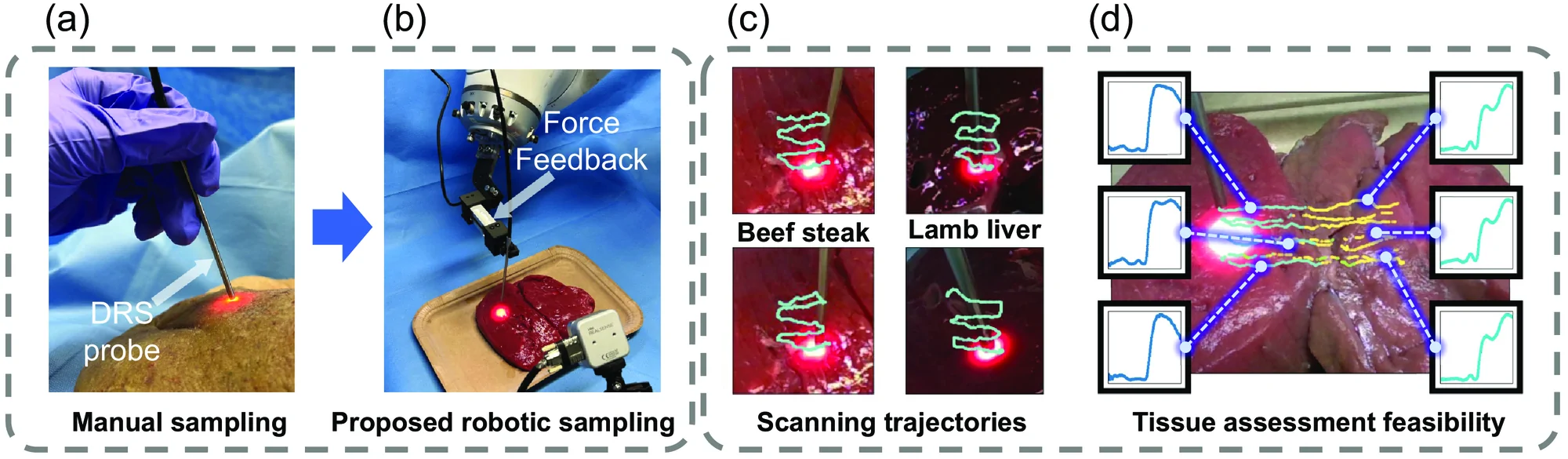
We present a robotic DRS scanning system with contact force regulation (b), designed to address key factors influencing DRS measurements. Like the manual sampling procedure (a), this system performs raster scanning over deformable tissue samples. The scanning trajectory is visualized in cyan (c). In a scanning experiment (d), the system successfully differentiates two distinct spectral regions within the tissue sample, with corresponding spectra displayed. These results highlight its potential as an effective tool for large-scale tissue assessment.

## 2 Problem Statement

### 2.1 Task Description

The sampling of DRS on a target specimen surface involves approaching the tissue and performing in-contact scanning. The probe must maintain consistent contact and pressure, which affects DRS measurement quality as shown in Fig. 2. This present challenges due to the difficulty in observing contact state while controlling minimal required force to prevent tissue injury. The DRS spectrum collected along the scanned path is recorded during the in-contact scanning stage. For robotic sampling, the grid-coverage path is often used to sample the target area which can be indicated by the surgeon on the monitor of a third-person view camera image. The probe was set to point downward, and only translational robotic motion was used. This is because these samples (such as *ex vivo* oesophagus tissues) are usually sufficiently flat when placed on the sampling plate.

**Fig. 2:**
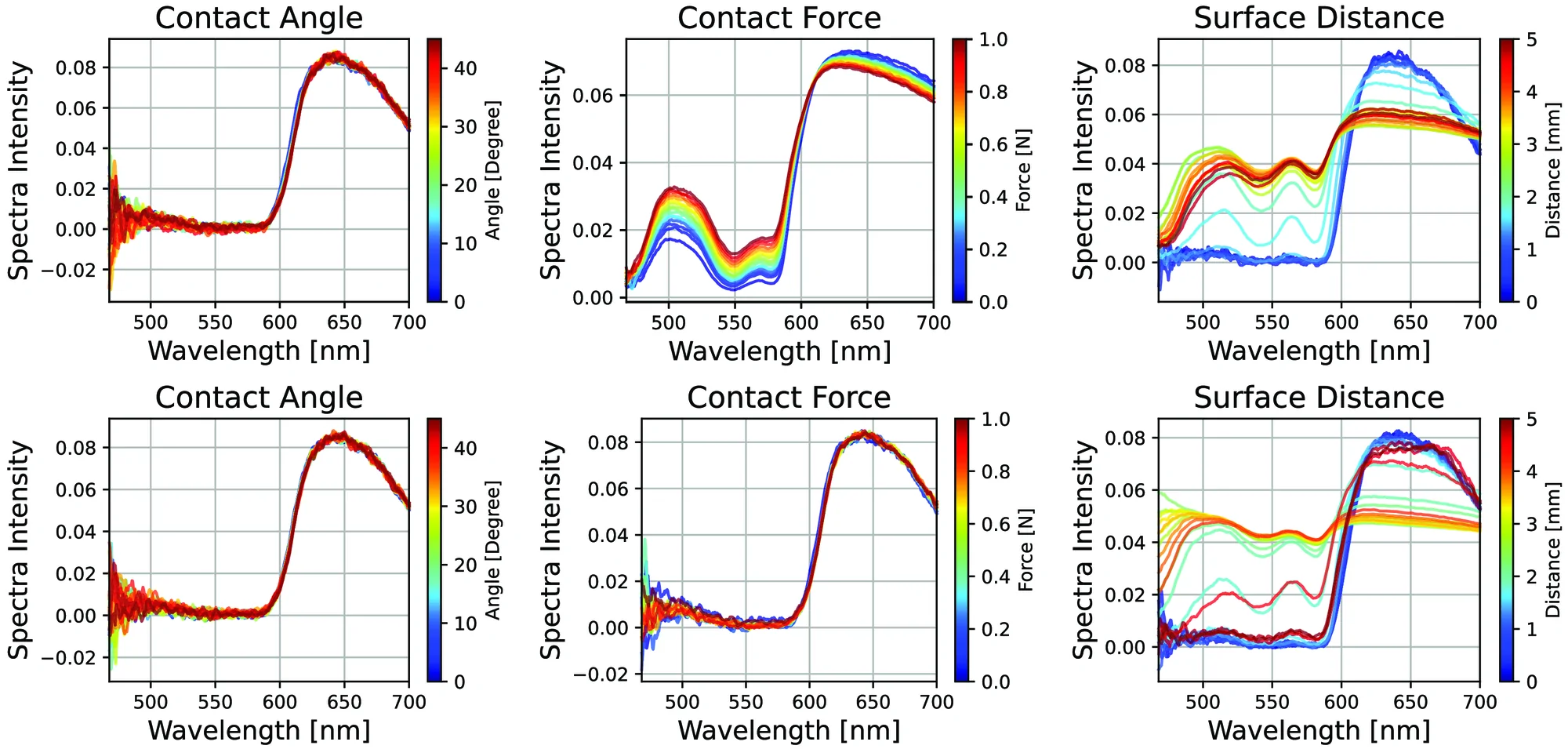
Visualization of the effects of contact angle, contact force, and surface distance on the beef sample. The contact angle is defined as the deviation between the probe orientation and the surface normal. Surface distance is defined as the gap between the probe tip and the sample surface, measuring zero upon contact. The two rows show spectra from two different sampling points.

### 2.2 Spectrum Evaluation

The reflectance spectrum of DRS can be denoted as $\mu_{\mathrm{DRS}}$. The quality of the DRS spectrum is closely related to the performance of the system. The spectral fingerprint (μ) indicates the composition of the target. It is the reflectance intensity received for each wavelength. These two components can be represented as

$$\mu (\lambda )=\frac{\mu_{\mathrm{DRS}}(\lambda )}{\|\mu_{\mathrm{DRS}}(\lambda ){\|}_{2}},$$ (1)

where λ is the channel of a specific wavelength. Thus, the spectrum sampled by manual operation and automatic robotic operation can be denoted as μ[M] and μ[A].

### 2.3 Objectives

The robotic scanning system is designed to achieve the following objectives while sampling: 1) regulate the probe contact force to ensure safe and consistent sampling, 2) minimize variance in the collected DRS data, 3) demonstrate the capability to distinguish sampled regions based on DRS data.

## 3 Method

### 3.1 Robotic Equipment Design

We utilized a KUKA LBR iiwa14 R820 robot arm for this system. The DRS instrument was mounted on the robot with a custom-fit 3D-printed holder and consisted of a handheld reflection fiber housed within a stainless steel probe, a mounting clamp, a halogen light source, a visible-near-infrared spectrometer, a load cell, and a data acquisition laptop. A RealSense D405 camera was fixed to the side of the robot acting as a third-person camera for visual servoing control. A parallel-beam load cell (Qisheng Sensor Co., China), capable of measuring forces up to 2 kg with a resolution of 0.01 g, was interfaced with a four-channel RS-485 load-cell transmitter. It was configured to measure force along the probe direction with a mounting shown in Fig. 3. The system was integrated using ROS and iiwa_stack was employed to command the KUKA robot.^22^ The overall system architecture is shown in Fig. 3.

**Fig. 3:**
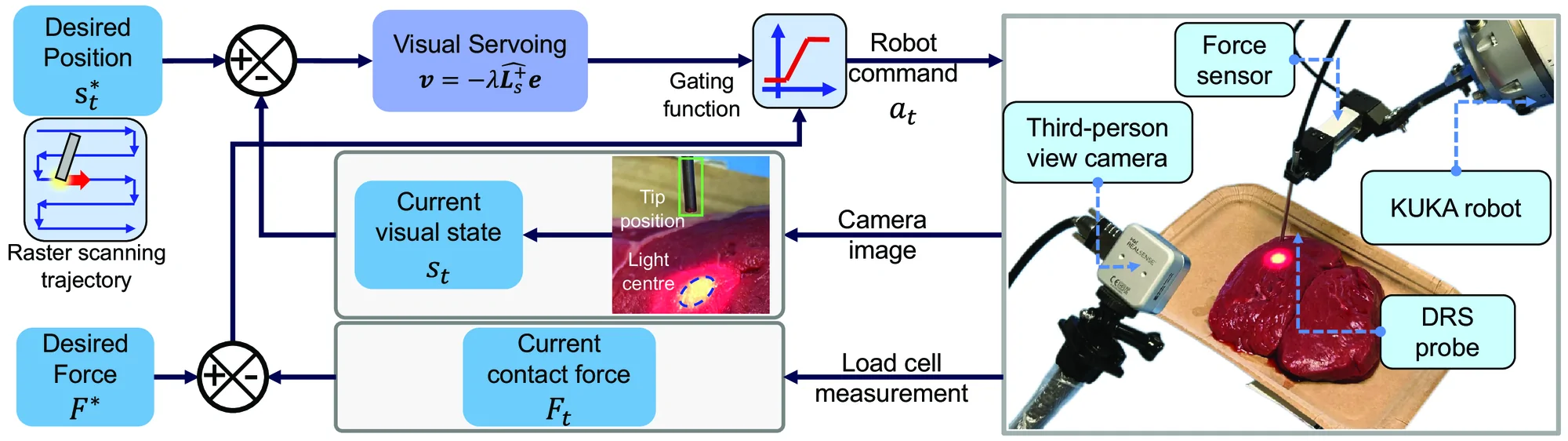
Overview of the control loop. The system follows a predefined grid coverage trajectory while maintaining a constant contact force. The current visual state is extracted from the camera view while the current contact force is measured from the load cell. A visual servoing controller minimizes visual state errors to track the desired position, with its output weighted and combined with force-tracking actions. The final robot command controls the robot to execute the scanning.

### 3.2 Visual Servoing

Image-based visual servoing (IBVS) was used to provide closed-loop control of the end-effector. It can improve the flexibility and robustness of the proposed scanning framework in clinically realistic environments. IBVS enables trajectory specification and adjustment directly in image space according to visible anatomical landmarks, thereby supporting more intuitive workflow integration and reducing reliance on precise robot calibration.^20^ In addition, the closed-loop visual feedback provided by IBVS improves robustness against variations in robot configuration, camera viewpoint, and cumulative positioning errors during large-area scanning, which may facilitate deployment in less structured surgical environments.

This control strategy was implemented during two critical phases: the approach phase, where the probe establishes initial contact with the tissue surface, and the scanning phase, where the probe maintains gentle contact while traversing the target area. IBVS aims to minimize the distance error of image features from their current position to the target position by iteratively approaching it through many small robot movements.^23^ This system refines the IBVS approach, building upon recent advances in automated tissue manipulation.^20^

This IBVS system used two pairs of image features consisting of the position of the probe tip and the centre of the illuminated light area on the global camera images. The error e represents the image plane vector pointing from the probe tip and the centre of the illuminated area to the given target point. This is the distance from the probe tip to the target sampling point. The velocity of the image features is represented by its derivative $\dot{s}(t)$. The Cartesian velocity of the end-effector is annotated as $\dot{x}$. The relationship between image feature space and Cartesian space is as $\dot{s}=J_{s}\dot{x}$, where $J_{s}$ is the image Jacobian. Thus, a closed-loop velocity controller to ensure an exponential decrease of the error $\dot{e}$ could produce a velocity command expressed as

$$v=-\lambda J_{s}^{+}e,$$ (2)

where λ is a positive gain and $J_{s}^{+}$ is the Moore-Penrose pseudo-inverse.

The position of the probe tip was detected using a YOLO-based detection module trained on *ex vivo* DRS sampling in the operating theatre.^24^ The centre of the illuminated light area was tracked from the segmented mask using a customized UNet^25^ with a ResNet-34^26^ encoder, which has been trained on bench-top sampling videos. Compared with rule-based classical computer vision approaches, the neural network–based method demonstrated superior precision and robustness across varying conditions. This segmentation model can reach a Dice score of 0.915 and a Jaccard score of 0.845.

Although it performed well on the test dataset, empirical evaluation in real-world setups revealed that the illuminated area could occasionally be missed, particularly in extreme cases after contact was established, as illustrated in Fig. 4. This failure often occurs due to the high absorption of light by the tissue sample during the scanning stage, which causes the tracker to be confused by reflections from ambient light. To enhance the robustness of the system, two features are cross-validated to prevent unexpected behaviour when any of them is lost or falsely detected. An empirical study showed that probe tip detection is more reliable than illuminated area tracking. Consequently, if the detected light centre deviates significantly from the probe tip position, only the more reliable probe position is preserved and duplicated for IBVS calculations, which requires two feature pairs. The system was programmed to scan the surface using a raster scanning path within a given rectangular area, which consists of a group of horizontal lines.

**Fig. 4:**
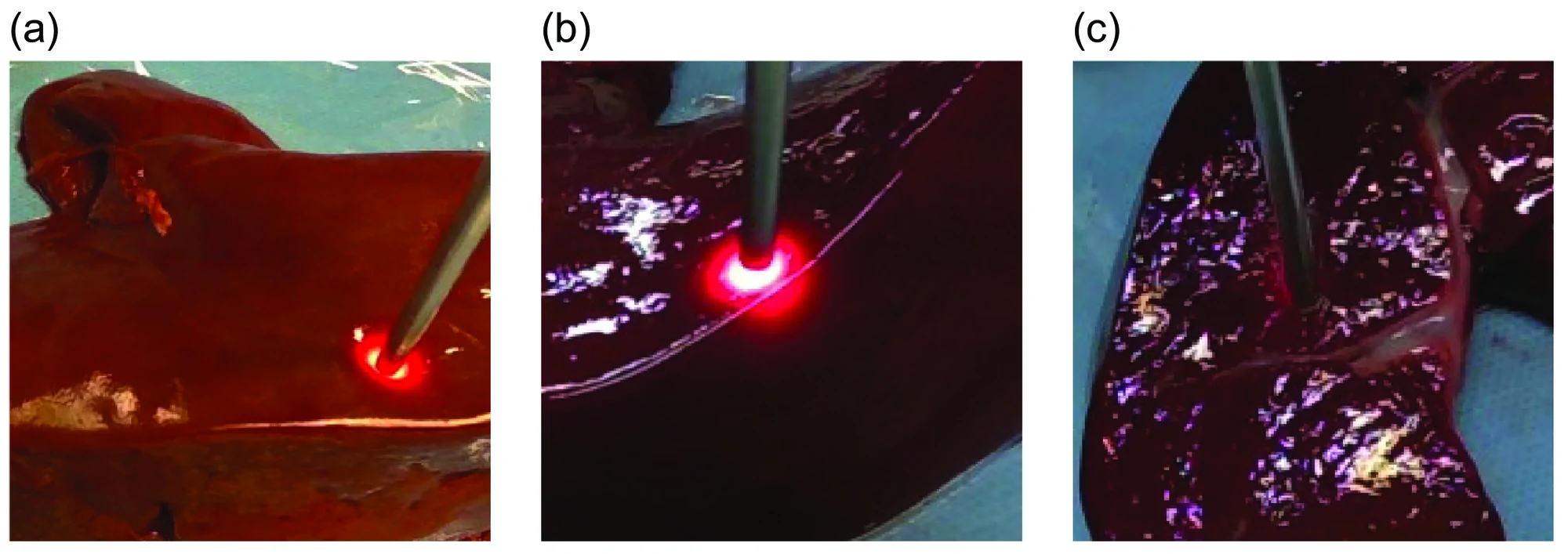
Examples of illuminated contact areas: three representative cases showing the appearance of the illuminated area after contact is established. In cases (a) and (b), the illumination appears as a bright circular ring around the contact point, exhibiting strong contrast against the reflected ambient light. However, in case (c), the illumination is significantly weaker and less discernible compared to the surface reflection of ambient light.

### 3.3 Contact Force Tracking

Contact force is one of the most important factors to guarantee safe measurements. In this system, only normal contact force along the vertical direction was measured. Targeted *ex vivo* samples investigated in previous works^2^^,^^6^^,^^7^ were sufficiently flat without significant inclination when placed horizontally on the examination platform. Therefore, the normal force dominates the contact interaction, hence also minimising lateral forces during DRS sampling. This approach ensures proper tissue interaction while maintaining focus on the primary control parameter.

An external load cell was used for probe tip force measurement because preliminary calibration experiments showed that the robot internally estimated force lacked sufficient precision in the low-force regime required for reliable force-regulated tissue interaction. The load cell can measure force in real-time at 120 Hz, enabling close tracking of the force changes during sampling. To ensure accurate contact force measurement, the system was zeroed before each execution to eliminate probe cable gravity effects. Comparison with scale measurements confirmed force differences within ±0.02  N, allowing us to disregard these minor variations. To mitigate high-frequency noise while preserving signal integrity, the raw data stream was processed using a cascade of a median filter (window=7) and a moving-average filter (window=5). This filtering strategy introduced only a minimal phase delay, which was inconsequential for the downstream control loop operating at 30 Hz.

The visual servoing motion and force-tracking motion were mixed by a dynamic gating function. This facilitates a balance between the close contact force control and the scanning speed. The gating weight is determined by the current measured force: $\beta =\max(\alpha_{\min},\min(k\times d+\alpha_{\min},\alpha_{\max}))$, where $\alpha_{\min}$ and $\alpha_{\max}$ are the lower and upper limit of the weight for visual servoing, $d=F(t)-F^{*}$ is the force tracking error to the target force $F^{*}$, and k is a parameter to modulate the blending of the two control modes. Therefore, the resulting action for mixing visual servoing $a_{\mathrm{VS}}$ and the contact force tracking $a_{\mathrm{FT}}$ became $a=(1-\beta )a_{\mathrm{VS}}+\beta a_{\mathrm{FT}}$. This gating mechanism can effectively prevent large contact force.

## 4 Experiments

### 4.1 DRS Equipment and Scanning Task Setup

Before each use, the spectrometer was calibrated using a standard Spectralon reflector to obtain the white $\mu_{W}$ and dark field $\mu_{D}$ reading, recorded with the light source on and off, respectively. The reading was thus normalized as $\mu_{\mathrm{DRS}}=\frac{\mu_{R}-\mu_{D}}{\mu_{W}-\mu_{D}}$ given the raw signal reading $\mu_{R}$. A Savitzky–Golay filter with a window length of 15 and polynomial order of 3 was subsequently applied to $\mu_{\mathrm{DRS}}$ for noise reduction while preserving higher-order spectral features. The visible light wavelength range from 468 nm to 720 nm of $\mu_{\mathrm{DRS}}$ was selected to represent the sample spectrum while other ranges were excluded due to the low signal-to-noise ratio. For post-processing of the collected spectrum, a frequency-domain filtering approach was applied to remove noisy spectra.

The control parameters for the IBVS and force-tracking controller were systematically tuned using an ovine hepatic tissue specimen to achieve optimal scanning performance. A target contact force of 0.08 N was selected to balance probe-contact stability against excessive tissue deformation, thereby ensuring signal consistency and spectral quality. The tuning procedure was conducted in three sequential stages. First, the visual servoing gain λ and the PID parameters for position control were calibrated. This was performed by disabling the force-tracking module (setting the gain β) and adjusting the parameters until the end-effector achieved a consistent free-space translational velocity of approximately 5  cm/s. Second, the target force setpoint $F^{*}$ and the PID parameters of the force-tracking module were tuned. This step involved initiating contact from a starting position 3 cm above the target tissue surface, constraining movement to the vertical axis. The parameters were adjusted to ensure a stable steady-state contact force of 0.08 N was reliably maintained for the best performance on the examined tissue. Finally, the gating weights of force gain $\alpha_{\min}$, $\alpha_{\max}$, and k were optimized. The objective was to enable stable and active scanning across the tissue surface while maintaining a target velocity of roughly 5  cm/s. The empirically determined ideal values from this tuning process were $\alpha_{\min}=0.5$, $\alpha_{\max}=0.8$, and k=4.

### 4.2 System Evaluation

This experiment aimed to address the following questions: 1) Can the system scan the sample safely? 2) Is the scanning force effectively regulated? 3) How consistent is DRS scanning when performed by the robot? 4) To what extent can the system enhance machine perception using DRS?

#### 4.2.1 Safety

The first experiment was to evaluate whether the system could successfully perform a scan while maintaining sample integrity. Critical safety parameters included preserving surface morphology without excessive penetration forces or shear stresses that could compromise tissue structure. The system safety has a predominant importance as this is one of the main objectives of this framework. System executed area scanning on two biological samples: ovine hepatic tissue and bovine muscle tissue, both obtained from a local store. The region of interest was selected from a homogeneous region on each sample. They were located on the parenchymal surface of the liver and the longitudinal muscle fiber surface of the bovine muscle tissue. The scanning was repeated 13 times per sample before the meat was affected by dehydration or minor plastic deformation from repeated scanning. The target contact force of the controller was set to 0.08 N to ensure optimal contact conditions without damaging the tissue. Other parameters were adjusted based on force tracking performance and scanning speed. A successful scanning was defined as completing the scanning with a force less than 0.5 N with no visible damage to the tissue sample. Following robotic scanning, an experienced DRS operator manually sampled the target area with a DRS probe to obtain a reference spectrum.

#### 4.2.2 Force regulation

The second experiment aimed to compare the applied force between robotic and manual scanning. An experienced DRS operator used the DRS end-effector to sample the ovine hepatic tissue, with the DRS spectrum and contact force recorded as a reference for comparison. An ablation study with contact force regulation disabled was also conducted to evaluate its effect on system behaviour.

#### 4.2.3 Spectral consistency

The third experiment was to compare the differences between DRS data collected manually and robotically. Six participants, all without prior experience in DRS sampling, were recruited to collect DRS spectra from the bovine muscle tissue. This cohort was chosen because the DRS instrument is a research prototype that has not yet been used in clinical practice, and its operation does not require specialized surgical skill or established expert techniques. Therefore, novice operators provide a valid baseline for evaluating the robot’s ability to standardize the sampling procedure. For the manual sampling, participants held the robot’s end-effector, detached from its flange, which incorporated an embedded load cell for contact force measurement. Participants practiced for five minutes following the provided instructions. Each participant sampled the selected region twice, with each attempt lasting ∼30  s. The spectral angle and root mean square error were used to evaluate the difference between the two spectra fingerprints $\mu_{a}$ and $\mu_{b}$ as follows:

$$\theta (\mu_{a},\mu_{b})={\;\cos\;}^{-1}(\frac{\mu_{a}\cdot \mu_{b}}{\left\|\mu_{a}\|\|\mu_{b}\right\|}),$$ (3)

$$\mathrm{RMSE}(\mu_{a},\mu_{b})=\sqrt{\frac{1}{N_{\lambda }}\overset{N_{\lambda }}{\underset{i=1}{\sum }}(\mu_{a}(\lambda_{i})-\mu_{b}(\lambda_{i}){)}^{2}},$$ (4)

where $N_{\lambda }$ is the total number of channels.

#### 4.2.4 Machine perception

The fourth experiment evaluated the capability of the DRS scanning system in visualising subtle spectral features on the sample surface. A bovine muscle tissue sample exhibiting two visually distinct regions with subtle colour differences served as the biological tissue model for this assessment. It was flatly placed on the platform with muscle fibers oriented orthogonal to the scanning surface. The robotic system executed systematic DRS measurements using a grid-coverage scanning pattern over a rectangular area (5  cm×3  cm) across both regions to acquire and classify spectral data. To ensure experimental consistency, four independent trials were conducted on the same sample under controlled conditions, maintaining uniform surface topology, minimising mechanical deformation effects, and preserving consistent oxygen and humidity levels throughout data acquisition.

Spectral classification was implemented using a support vector machine (SVM), a widely-used binary classifier in classifying DRS spectra,^27^ trained on pre-collected DRS reference spectra from each region. All spectra were labelled into a binary class that describes whether they are sampled from one specimen or the other. Totally 1605±45 spectra were collected for each test case. The classifier was trained with DRS samples pre-collected in two regions with 50 data points each before being used to classify these scanning-collected spectra. To minimize variance within each class and to construct a controlled test scenario, the training and evaluation were restricted to these two well-characterized regions, which are spatially related. The classifier operated with a confidence threshold of 70% to distinguish between the two tissue regions based on their spectral signatures. Classification accuracy was evaluated by the correct assignment of individual spectral measurements to their corresponding tissue regions. Successful autonomous tissue assessment was defined as the generation of a spectral classification map that accurately delineated the two distinct regions, demonstrating the system’s capacity for real-time spectral analysis and tissue differentiation.

## 5 Results

### 5.1 Scanning Safety and DRS Analysis

To achieve our target of 13 successful scans, the experiments had to be conducted 13 times for the bovine muscle tissue and 14 times for the ovine hepatic tissue, yielding success rates of 100% and 93%, respectively, which is higher than a recently reported DRS system.^20^ The failed trial on the ovine hepatic tissue was interrupted due to specular reflections from ambient light interfering with the detection of the illuminated area. The light-leakage problem mentioned in the previous system^20^ has also been mitigated as a firm contact can be established during the scanning. The system was further tested on another bovine muscle sample, running continuously for ∼30  min, including execution and resetting. All 46 trials across different sampling areas were completed successfully without tissue damage or system malfunctions. Representative recordings of the sampling process are provided in Video 1. The scanning trajectories were generated using a raster-scanning pattern comprising five parallel horizontal linear paths of varying lengths defined in image space. The corresponding physical scanning areas on the tissue surface varied between cases, ranging from 1 to $4\;{\mathrm{cm}}^{2}$, dependent on the area scanning strategy. The average scanning coverage speed was $14.3\;{\mathrm{cm}}^{2}/\min$ with an average linear scanning speed of 5.52  mm/s with a variance of 1.18  mm/s. In most cases, the scanning was finished within 1 min, excluding the approaching stage (between 2 and 10 s). During scanning, shear deformation caused minor but visible changes in the bovine muscle tissue; however, no noticeable spectral variation or instability attributable to tissue deformation was observed throughout the experiments. The DRS data collected from robotic scanning, μ[A], are shown in Fig. 5 and compared with the manually collected data, μ[M]. The mean spectra of μ[A] generally align with those of manual scanning but exhibit slightly higher variance.

**Fig. 5:**
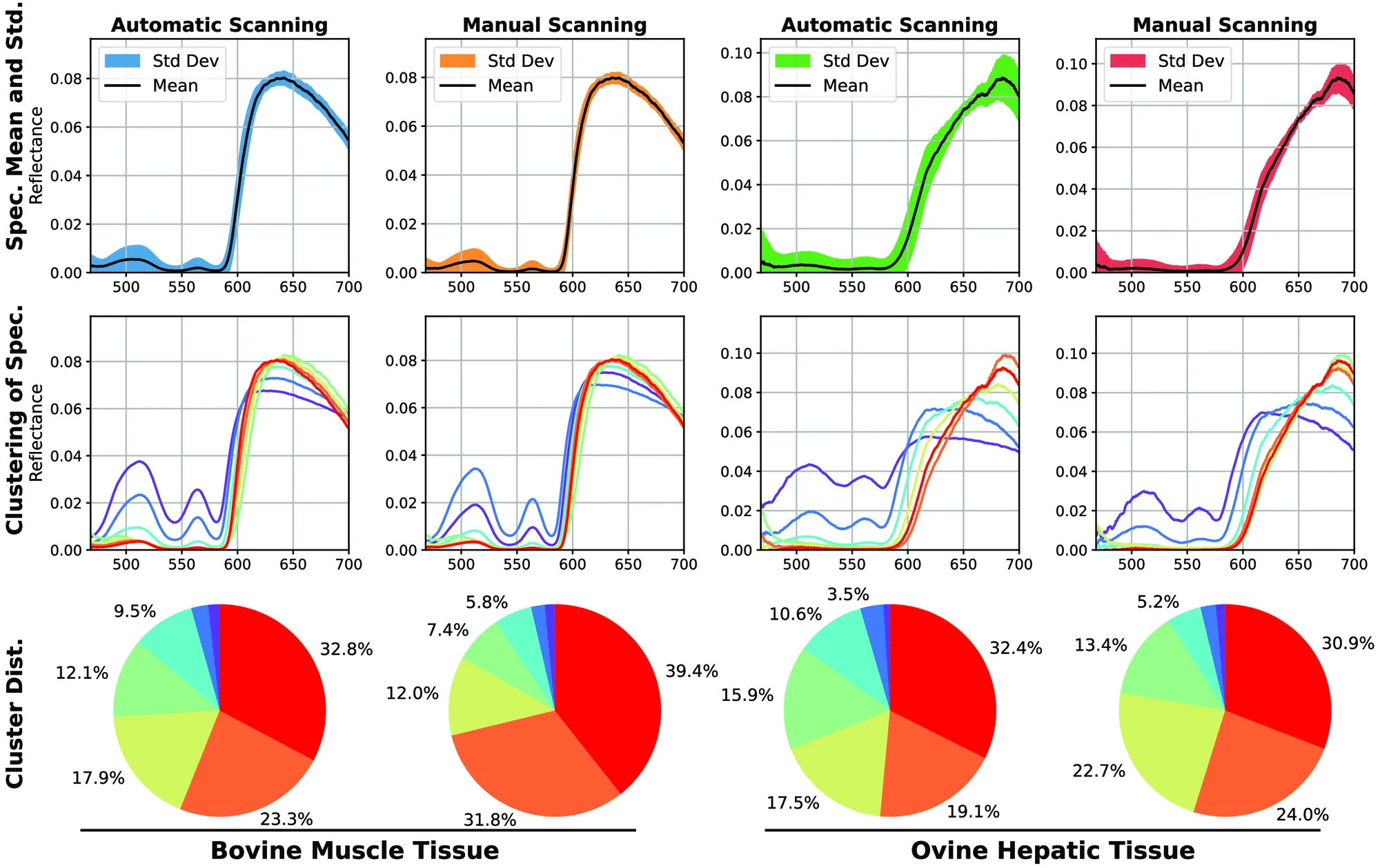
Visualization of collected spectra. On each row, four groups of spectra are included for automatically and manually collected spectra on bovine muscle tissue and ovine hepatic tissue. The top row shows the mean and standard deviation of each group of spectra. The vertical axis is reflectance rate, and the horizontal axis is wavelength [nm]. The middle row shows the clustering results of each spectra group by showing the centroid spectrum of each cluster. The bottom row shows the pie charts of clustering, indicating the ratio of spectra that belong to that cluster, with colours matching the centroid spectra in the middle row. Proportional distribution values of cluster components comprising less than 3% of the total is omitted from the pie charts.

Spectral composition analysis was performed using clustered spectra to characterize the overall properties of the acquired dataset. Given that DRS measurements exhibit inherent noise due to high sensor sensitivity and tissue heterogeneity, clustering analysis was applied to identify representative spectral patterns and reduce data complexity for systematic evaluation.

We employed the K-Means clustering algorithm with seven clusters to group spectra with similar characteristics to simplify dataset interpretation. It should be noted that this clustering analysis serves as an exploratory tool for characterizing spectral data quality and system performance, rather than for clinical tissue discrimination. The selection of seven clusters was based on empirical optimization, providing sufficient granularity to distinguish unique spectral signatures while maintaining manageable complexity for the experimental analysis. The cluster centroids of the spectrum and the proportion of spectra in each cluster are as illustrated in Fig. 5.

Notably, the distribution of spectrum centroids in the bottom row exhibits greater similarity in bovine muscle tissue compared to ovine hepatic tissue. In the ovine hepatic tissue scans, a higher proportion of spectra deviated from the most frequently presented spectrum. The clusters were ranked based on the ratio of spectra in each cluster. In the three least represented clusters, which contained the most deviated spectra, 15.1% of μ[A] fell into this group, compared to 9% in μ[M]. In addition, the purple line represents a flatter spectrum than the others, potentially indicating specular reflection on the surface. This suggests that robotic scanning may introduce outlier spectral signals.

### 5.2 Contact Force Comparison

The distribution of the detected force for both the robotic system and an experienced operator is illustrated in Fig. 6. The mean force values are 0.080 N and 0.079 N, and the 95th percentile force values are 0.217 N and 0.177 N for manual and robotic sampling, respectively. The applied force in robotic sampling closely matched that of manual scanning, with a lower variance of 0.055 N compared with 0.069 N.

**Fig. 6:**
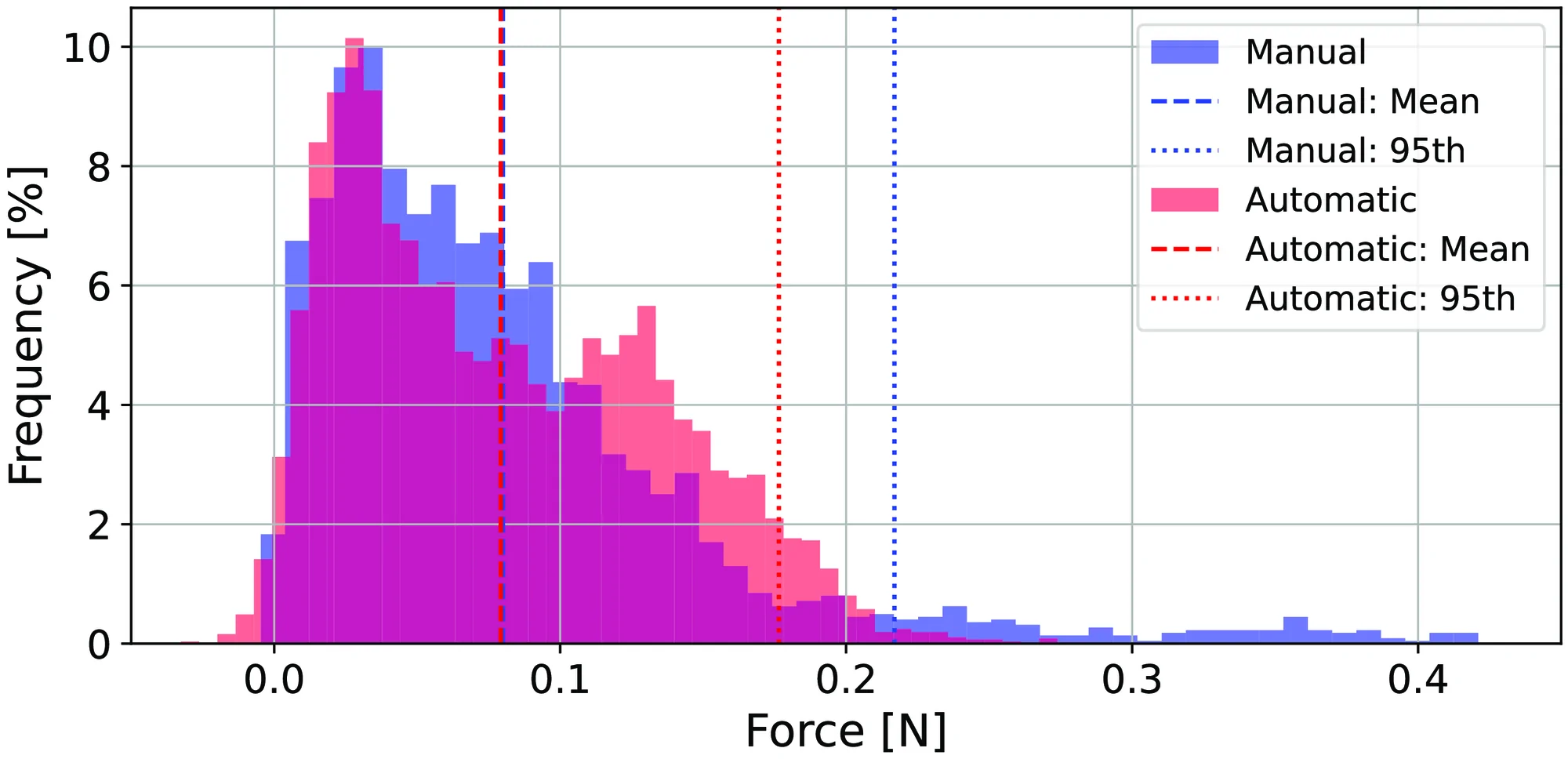
Comparison of contact force distribution during manual and automatic sampling. The mean and 95th percentile force values are marked using dashed lines. The overlapping area is shaded in purple.

Force values below zero shown in Fig. 6 could be attributed to the tension from bending the optical cable or the drift in the force sensor signal during the measurement. Furthermore, the system effectively maintained the contact force below 0.3 N, ensuring a safe sampling process for delicate tissue samples.

Empirical evidence indicates that applying 0.3 N of force, equivalent to 37.3 kPa, preserves specimen integrity. This aligns with prior research in which a probe-based contact detector applied a maximum force of 0.3 N and pressure of 77.4 kPa in liver tissue experiments.^28^ To summarize, the analysis of the applied force tracking reveals that the system can avoid extreme force values better than human operators, thereby ensuring tissue integrity during scanning.

#### 5.2.1 Ablation study

Directly disabling contact force regulation resulted in excessive contact force immediately upon tissue contact, leading to visible tissue damage and unstable scanning behaviour. This experiment has been repeated for three trials on both bovine and ovine tissue samples, leading to consistent results. Due to these safety concerns, contact force regulation should remain enabled throughout the scanning process. This finding highlights the critical role of contact force regulation in ensuring safe and effective robotic DRS scanning, particularly when interacting with delicate biological tissues.

### 5.3 Consistency on DRS

Interobserver and intraobserver variability indicate the consistency of sampled DRS with the same and different observers. These properties were evaluated on the entire collected spectrum dataset μ[k] where k∈K={1,…,6,A}, which comes from six operators (μ[M:I] to μ[M:VI]) and the robotic system μ[A].

#### 5.3.1 Interobserver variance

This was assessed by comparing spectral similarity between each participant and all others, including all human operators and the robotic system. Table 1 presents the overall mean values for each sampler as Mean(θ(μ[i],μ[j])) and Mean(RMSE(μ[i],μ[j])) for i,j∈K with i≠j. Results indicate that the robotic system showed a subtly higher variance compared with human operators. However, its performance metric of 1.43 deg remained clinically acceptable and comparable to 1.28 deg from Operator I.

**Table 1:** Interobserver DRS variance.

Metric	Operators						Robot
	I	II	III	IV	V	VI	
θ**[°]**↓	1.280	0.769	0.987	0.768	1.221	1.068	1.431
**RMSE**↓	1.034	0.623	0.799	0.622	0.987	0.863	1.159

θ is the average spectral angle difference from one sampler to all others (including all operators and the robot), measured in degrees. RMSE is the average Root Mean Square Error to other operators.

#### 5.3.2 Intraobserver variance

This was assessed by measuring the dispersion of the spectra relative to their own mean value. As shown in Table 2, μ[A] results in subtly higher dispersion levels in terms of mean spectral angle and root mean square error. However, the variance in robotic scanning remains comparable to that of human operators.

**Table 2:** Intraobserver DRS variance.

Metrics	Operators							Robot
	I	II	III	IV	V	VI	All	
θ**[°] Mean** ↓	3.282	3.244	2.844	3.241	3.462	3.169	3.220	4.447
θ**[°] Median** ↓	2.576	2.553	2.408	2.414	2.565	2.553	2.511	3.299
θ**[°]P85** ↓	4.546	4.595	3.670	5.223	6.075	4.666	4.765	7.579
**RMSE**	3.398	3.335	2.825	3.332	3.313	3.106	3.284	4.478

θ is the spectral angle difference from all individual spectra to the mean spectra of a spectral group, measured in degrees. Mean, Median, and P85 represent the mean, median, and 85th percentile of all spectral angles. RMSE is Root Mean Square Error using $10^{-3}$ as the base.

#### 5.3.3 Result interpretation

These findings indicate that the contact conditions of robotic sampling exhibit slight variations compared with manual collection. This discrepancy may be attributed to differences in contact force management, as human operators can precisely control force in three dimensions, whereas the robotic system is limited to one-dimensional force regulation. However, these variations are acceptable within the context of tissue classification. Previous research on DRS^7^ has reported spectral differences of up to 10.0 deg and 19.8 deg between cancerous and healthy regions in oesophageal and gastric tissues, respectively. Given this substantial margin, the minor discrepancy between robotic and manual collection is unlikely to affect tissue differentiation. Therefore, the system remains suitable for this application.

### 5.4 Visualization of Spectral Change

The visualization of the DRS scanning results for a bovine muscle tissue with two distinct regions is shown in Fig. 7, while the complete process is demonstrated in Video 1. The predicted probability of the tissue class (either from the left or right) at each scanning waypoint is overlaid on the image. The mean classification accuracy for each region across four trials reached 93.8%. However, a false negative prediction can be observed in case (c). This may be attributed to minor heterogeneity in that area, which could cause subtle variations in the DRS spectra.

**Fig. 7:**
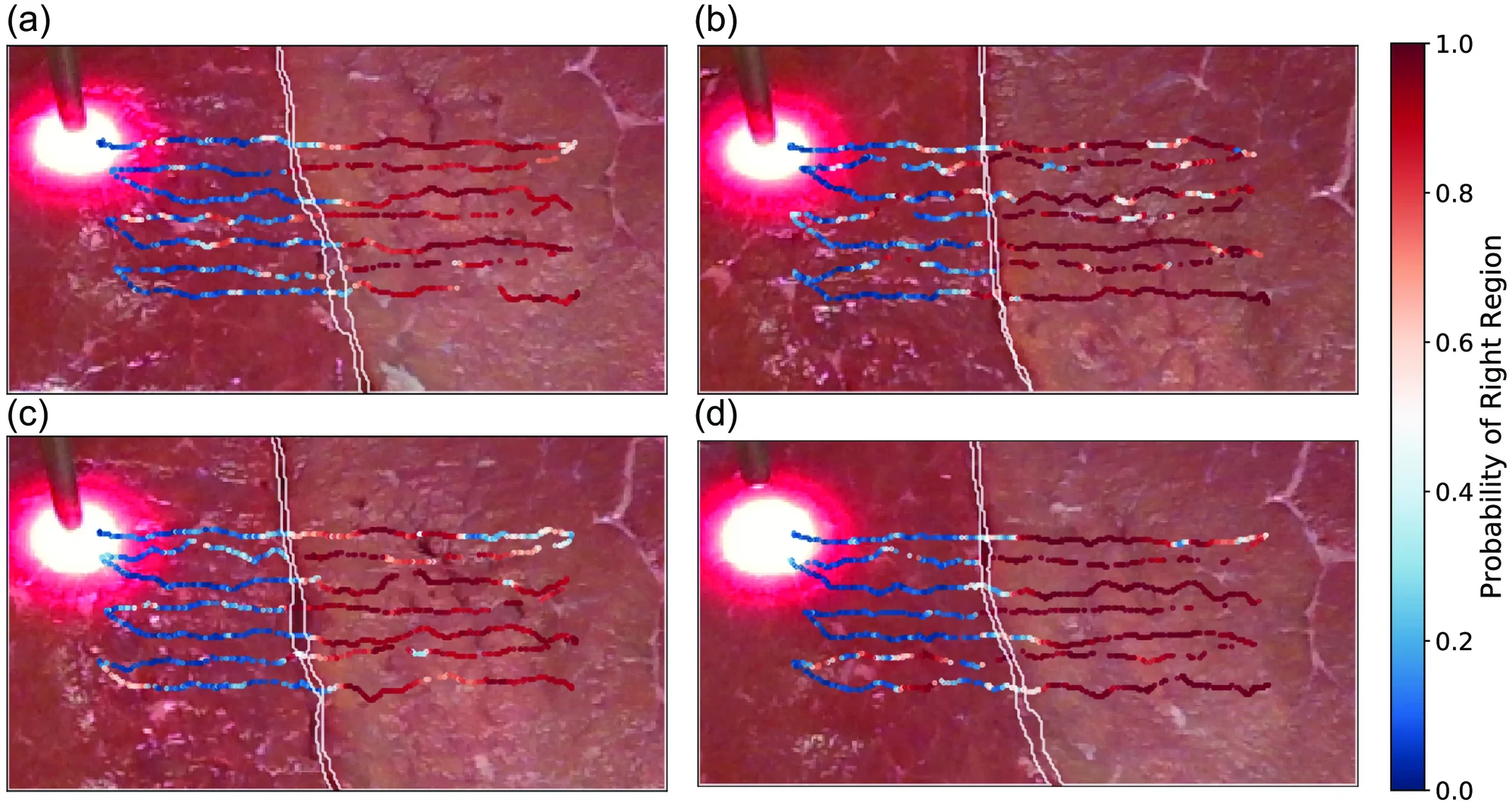
Visualization of DRS-based tissue classification results (Video 1, MP4, 7.6 MB [URI: https://doi.org/10.1117/1.JBO.31.12.123306.s1]): DRS spectra were used to distinguish between two visually similar tissue regions shown in the background of each panel. A trained classifier predicted the tissue source for each spectrum acquired along the scanning trajectory. The scanning path is colour-coded according to predicted classification probability, with blue indicating the left tissue region and red indicating the right region. The transparent grey line at the centre denotes the boundary between the two tissue samples.

Occasional spectral deviations that do not conform to either class may correspond to fascia or other anatomical structures. As this sample is a cross-section of muscle fibers, such structures may locally alter the DRS spectra. Nevertheless, these minor spectral alterations are unlikely to affect the performance when applied to clinical tissue classification tasks with more pronounced spectral differences—such as distinguishing cancerous from healthy tissue, as discussed earlier. Thus, this result highlights its capability to differentiate tissue regions based on spectral characteristics, demonstrating its potential for automated tissue classification.

## 6 Discussion

### 6.1 Advantages Over Current Gold Standard

The current gold standard for intraoperative tissue assessment, frozen section analysis, requires 20 to 30 min on average, prolongs operative duration, exhibits significant inter-operator variability, and is limited to sampling small tissue areas.^29^^,^^30^ In direct comparison, the proposed robotic DRS system completed scanning of a representative tissue region in approximately one minute, representing an order-of-magnitude reduction in tissue-interrogation time relative to frozen section analysis. Although assigning a clinical practitioner to assess *ex vivo* samples using the DRS offers an alternative, this approach requires an additional skilled operator for system operation and data acquisition. Because the proposed system performs scanning autonomously, it further reduces the need for dedicated personnel, whereas frozen section analysis relies on trained laboratory technicians and pathologists. In contrast to these, an automated DRS system could deliver a preliminary assessment of a larger tissue area within minutes. Its operation would retain the integrity of the tissue sample, which does not affect the following tissue assessment procedure while integrated in the current workflow. This capability would prove particularly advantageous in oncological procedures requiring rapid intraoperative margin assessment, thereby enhancing surgical precision and completeness of tumor resection without compromising operative efficiency. Moreover, the robotic scanning speed is comparable to that of manual operation and therefore remains within clinically acceptable time constraints, while offering the potential for real-time tissue characterization as previously demonstrated for *in vivo* DRS measurements.^2^ Together with the demonstrated *in vivo* feasibility of the DRS probe, these characteristics position the proposed robotic DRS system as a promising candidate to become a novel intraoperative tool for tissue differentiation.

### 6.2 Influence of Tissue Mechanical Properties on Scanning Performance

Tissue deformation during scanning is an inherent consequence of the firm probe-tissue contact required for reliable DRS sampling. Two modes were observed: indentation from the normal contact force, and shear-related displacement from lateral probe motion. Indentation was well tolerated by both the bovine muscle and ovine hepatic tissues, with no discernible damage. However, shear-related displacement was considerably more pronounced in the compliant bovine muscle than in the stiffer ovine liver under equivalent conditions. The suitability of this approach therefore depends on the mechanical properties of the target tissue. In the intended clinical applications—gastric and oesophageal assessment—this is unlikely to pose a practical limitation, as these tissues have previously demonstrated stable contact behaviour during clinical validation.^2^^,^^6^ Furthermore, the desired contact force may be adjusted to optimize probe–tissue optical coupling while minimising tissue deformation during scanning.

### 6.3 Limitation of the Current Test Case

The example testing case mentioned in Secs. 4.2.4 and 5.4 may differ from the conditions encountered in real clinical environments. In this initial validation, the training and test sets were constructed using a random point-wise split of spectra acquired from a single pair of specimens, with the intention of accounting for spatially correlated spectra within each sample. Furthermore, the selected biological tissues were chosen to emphasize spectral differences under relatively controlled conditions, with limited intrinsic variability. Although this design supports a focused proof-of-concept evaluation, it may not fully capture the complex and often subtle spectral differences between malignant and benign tissues observed in routine pathological practice, and thus might not fully reflect the method‘s performance on heterogeneous clinical tissue samples. Future work will therefore include evaluations on specimens with diverse pathological subtypes and specimen-level train-test separations to more accurately assess the robustness and generalisability of the proposed approach in realistic clinical scenarios.

### 6.4 Future Work

Although one-dimensional force feedback successfully managed contact conditions, implementation of three-dimensional force sensing could further enhance measurement precision and inter-sample consistency through comprehensive contact characterization. Future efforts will prioritize validation on pathological specimens and seamless integration within established surgical workflows. Initial validation will employ animal tissue models with implanted phantom lesions to simulate pathological conditions in controlled laboratory environments. Clinical translation will leverage intraoperative *ex vivo* oesophageal and gastric tissues, taking advantage of DRS’s proven ability to detect structural and biochemical differences in gastrointestinal tissues.^2^^,^^6^ As standardized protocols for DRS-based intraoperative margin assessment have not yet been established for either *in vivo* or *ex vivo* applications, future work will also characterize the scan time required for clinically representative surgical margin areas and confirm that the resulting acquisition times remain within intraoperative workflow constraints. This progressive validation strategy will establish the system’s diagnostic performance across diverse tissue types while maintaining surgical workflow compatibility.

## 7 Conclusion

In this work, we presented an automatic robotic-assisted DRS scanning system combining visual servoing with precise force control. Our approach maintains contact forces below 0.3 N, substantially mitigating mechanical stress on biological tissues compared to manual operation. The capability of sensing contact force makes the system safer than the recent reported system,^20^ leading to an improved successful scanning rate. This lower force application helps prevent DRS pressure effect that could alter optical properties. Through comprehensive experiments, we evaluated spectrum collection procedures against manual methods, confirming the system consistently applies well-controlled forces while preserving sample integrity. By incorporating a DRS spectral classification algorithm, the system exhibits high-sensitivity tissue differentiation capabilities. Consequently, this work demonstrates the efficacy of the proposed system as a reliable, autonomous diagnostic assessment tool.

## Supplementary Material

10.1117/1.JBO.31.12.123306.s1

## Data Availability

The experimental data that support the findings of this article are available from the corresponding author upon reasonable request, subject to acceptance. The core computational code developed for this study is publicly available on GitHub: https://github.com/KDeng98/RoboticDRS
